# Up to “Me” or Up to “Us”? The Impact of Self-Construal Priming on Cognitive Self-Other Integration

**DOI:** 10.3389/fpsyg.2012.00341

**Published:** 2012-09-14

**Authors:** Lorenza S. Colzato, Ellen R. A. de Bruijn, Bernhard Hommel

**Affiliations:** ^1^Institute for Psychological Research, Leiden UniversityLeiden, Netherlands; ^2^Leiden Institute for Brain and Cognition, Leiden UniversityLeiden, Netherlands

**Keywords:** self-construal priming, SSE, self-other integration

## Abstract

The degree to which people construe their perceived self as independent from or interdependent with their social environment can vary. We tested whether the current degree of social self-construal predicts the degree to which individuals integrate others into their self-concept. Participants worked through tasks that drew attention to either personal interdependence (e.g., by instructing participants to circle all relational pronouns in a text, such as “we,” “our,” or “us”) or independence (by having them to circle pronouns such as “I,” “my,” or “me”) and were compared with respect to the social Simon effect (SSE) – an index of the degree to which people co-represent the actions of a co-actor. As predicted, the SSE was more pronounced in the interdependence group than in the independence group, suggesting that self-other integration varies dynamically as a function of the relative saliency of the other.

## Introduction

Western societies take it commonly for granted that people own some sort of “self,” a concept that refers to the phenomenal and social identity of a person over time and his/her responsibility for his/her actions. Eastern cultures are often more skeptical; e.g., Buddhism considers the self as only apparent and seeks to overcome it through systematic mental training (the anatta doctrine). Even though there is no agreed-upon definition of the concept (Neisser, 1988), authors often distinguish between what has been called the “minimal self” (Gallagher, 2000) and the “narrative/diachronic self” (Dennett, 1992). While the latter refers to the social identity people construct by actively creating their (ideally coherent) autobiography, the former refers, among other things, to the phenomenal experience that one has a body that is different from others’ and that can be employed to actively change one’s environment. How much that experience is fueled by, and thus depending on self-perception has been emphasized by Hume (1739/1978, p. 252): “when I enter most intimately into what I call myself, I always stumble on some particular perception or other, of heat or cold, light or shade, love or hatred, pain or pleasure,” an observation that led Hume to conceptualize the self as a bundle of perceptions (a construction that roughly corresponds to James’ concept of “me”; James, 1890). Hence, the cognitive system may represent oneself as just another event, that is, as an integrated network of codes representing one’s own perceptual features (Hommel et al., 2009). Along the same line, very recently, it has been shown that Buddhist practice, which is assumed to “remove the barriers between oneself and others” (Dogen, 1976, p. 39), which should lead to a loss of discrimination between the representation of oneself and the representations of others, enhances self-other integration (Colzato et al., 2012).

The present study tested whether the degree of self-other integration is not only determined by such slow learning processes but also depends on more situational, dynamic factors. Previous research suggests that the degree to which individuals perceive themselves as dependent on, or independent from their social environment might vary rather quickly. For instance, Kühnen and Oyserman (2002) showed that having participants to circle all relational pronouns in a text, such as “we,” “our,” or “us,” induces a global, context-sensitive processing strategy, while having them to circle pronouns referring to the self independent from others, such as “I,” “my,” or “me,” induces a local, context-insensitive processing strategy. Even though this observation does not prove that priming can produce long-lasting modifications of the basic structure of self-perception, it does suggest that task and context can temporarily affect people’s attention in such a way that they perceive themselves either as a part of a social context (as interdependent) or more in isolation (as independent). If so, one would expect that interdependence priming would lead them to integrate others into their own self-concept to a greater degree than independence priming. We assessed this hypothesis by testing whether self-construal priming modulates the social Simon effect (SSE; Sebanz et al., 2003).

The classical Simon effect shows that left and right actions are carried out faster if they spatially correspond to the stimulus signaling them (Simon, 1969). Recent studies revealed that this is the case even when the two actions are carried out by different people (i.e., the SSE), which has been taken to imply that task representations are socially shared (for overviews, see Sebanz et al., 2006). Very recently Hommel and colleagues (2009) provided evidence that the SSE occurs only if actor and co-actor are involved in a positive relationship (induced by a friendly acting, cooperative confederate) but not if they are involved in a negative relationship (induced by an intimidating, competitive confederate). Hence, the mere presence of another person is insufficient for the SSE to occur if this person is not involved in the task (Sebanz et al., 2003) or is perceived as intimidating and unfriendly (Hommel et al., 2009). This suggests that people consider the other person’s action in their own representation of the current task and that the SSE can be considered to indicate the degree to which the participant has integrated another person’s actions into his or her own task representation (Sebanz et al., 2003; Hommel et al., 2009). If drawing people’s attention to personal interdependence or independence affects the degree to which people integrate others into their own self-concept, one would expect a more pronounced SSE with the former than with the latter.

## Materials and Methods

Forty-four healthy young adults, with a mean age of 22.5 years (SD = 2.4, range 18–30), participated for partial fulfillment of course credit or a financial reward. Written informed consent was obtained from all participants after a detailed explanation of the study procedures. The protocol was approved by the local ethical committee (Leiden University, Faculty of Social and Behavioral Sciences).

**Figure 1:**
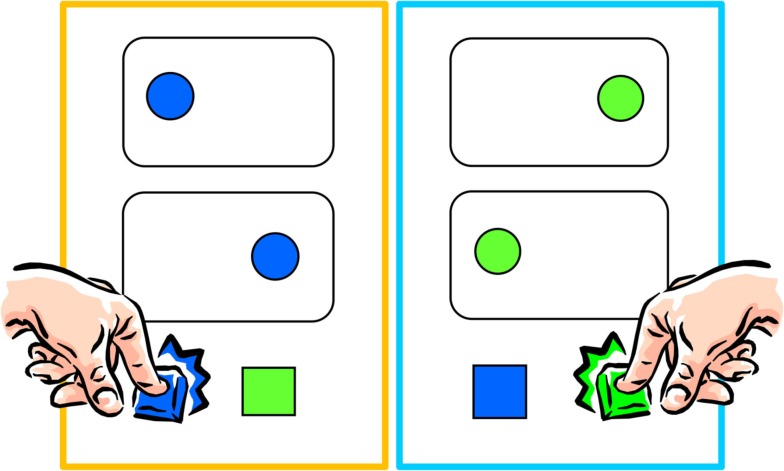
**Setting in the social Simon task: the task was distributed among two individuals**. Each person responded to only one of the two colors.

In the social Simon task participants made speeded discriminative responses to the color (green or blue) of circles by pressing one of two keys while the other key was operated by another participant (see Figure 1). Circles (diameter of 43 pixels) were equiprobably presented to the left or right (at a distance of 50 pixels) of a central fixation point (12 pixels) until the response was given or 1,500 ms has passed. Intervals between subsequent stimuli varied randomly but equiprobably, from 1750–2250 ms in steps of 100 ms. Participants were to ignore the location of the stimulus and to base their response exclusively on its color. Responses were to be given as fast as possible while keeping error rates below 15% on average; feedback about general speed (averaged between the RT of the two participants) was provided at the end of a trial block. The task consisted of one practice 60-trial block and three experimental 60-trial blocks. Just like in the original version of the task (Sebanz et al., 2003), the participants sat next to each other, attended to the same screen, and responded both with their dominant hand.

Eleven pairs of participants, randomly determined, were asked to constantly switch between circling the independent (e.g., I, mine) pronouns in an essay for 2 min (independent self-construal priming) and completing a block of the social Simon task. The other 11 pairs of participants constantly switched between circling the interdependent (e.g., we, ours) pronouns in an essay for 2 min (interdependent self-construal priming) and performing a block of the social Simon task. Given that the experiment was composed of one practice and three experimental blocks, participants were to switch between the prime and the probe task four times in total. Participants were naïve to the experiment. When debriefed after the study, all participants thought that the study was about cooperation. None pointed out the possible relation between the social Simon task and the fact they had to circle the relational pronouns.

## Results

A significance level of *p *< 0.05 was adopted for all tests. Mean reaction times (RTs) from correct trials and error rates were analyzed by means of ANOVAs as a function of Priming Group (independence vs. interdependence) as between-participants factor and spatial stimulus-response Correspondence (correspondence vs. non-correspondence) as within-participants factor.

**Figure 2:**
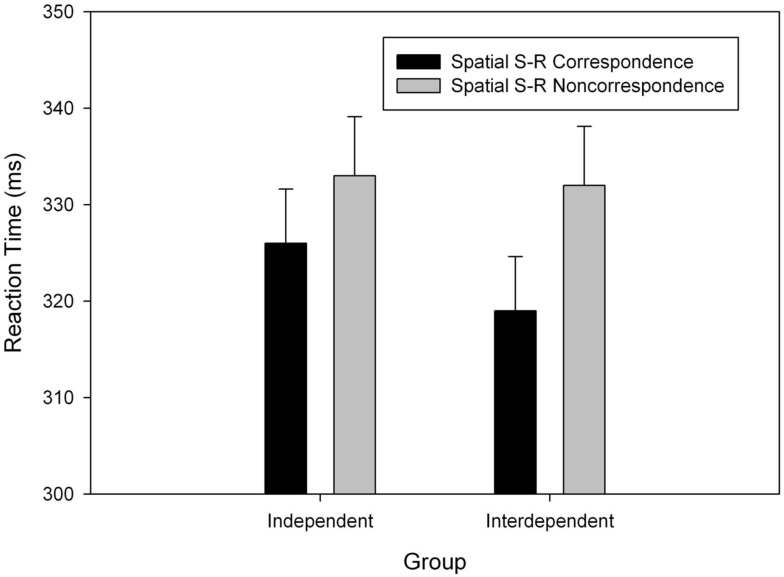
**Mean reaction time as a function of group (Independent vs. Interdependent) and spatial stimulus-response (S-R) correspondence**. Error bars show standard errors of the means.

The reaction time analysis showed no evidence of a group effect, *F *< 1, but a main effect of correspondence, *F* (1, 42) = 40.19, *p* < 0.001, MSE = 50.777, $\eta_{p}^{2}$ = 0.49, indicated that responses were generally faster with stimulus-response correspondence than with non-correspondence (322 vs. 332 ms). More importantly, a significant interaction indicated that the correspondence effect on RT differed between groups, *F*(1,42) = 4.65, *p *= 0.037, MSE = 50.777, $\eta_{p}^{2}$ = 0.10, Even though the correspondence effect was reliable in both, the interdependence, *F*(1,21) = 40.31, *p* < 0.001, MSE = 45.472, $\eta_{p}^{2}$ = 0.66, and the independence group, *F*(1,21) = 7.91, *p *= 0.010, MSE = 56.083, $\eta_{p}^{2}$ = 0.25, the SSE was significantly more pronounced in the interdependence group (see Figure 2). Moreover, follow-up analyses showed that the two groups did not differ in the corresponding trials and that the size of SSE did not change over time adding block as additional factor in the ANOVAs, *F’s *< 1.

Overall, error percentages on corresponding trials (0.06%) and non-corresponding trials (0.1%) were comparable and did not differ between Groups (*F’s *< 1).

## Discussion

As expected, the SSE was more pronounced in the interdependence group than in the independence group. This finding suggests that having people work through tasks that draw attention to either personal interdependence or personal independence affects the degree to which people integrate other people with their own self-concept.

Hommel et al. (2009) suggested the Theory of Event Coding (TEC; Hommel et al., 2001) as theoretical framework to explain the mechanism underlying the SSE. TEC assumes that both perceived events and produced events (i.e., actions) are cognitively represented by codes of their perceptual features (such as color and shape of objects, the sensory feedback and affective consequences of actions, etc.). Along these lines, other people can be considered as just another type of event, which would be cognitively represented by codes of the features that describe what the given individual looks like, which perceivable action effects he or she is currently producing, which affective states are triggered by this person, and so forth. And the same would hold for the perceiving person him- or herself: one might represent oneself, including one’s body parts, just as any other event and code oneself in terms of one’s perceptual attributes and perceivable action effects. Self-other integration is, then, assumed to be a function of the overlap between the features bound to, and thus constituting self and other.

From this perspective, independence priming along the lines of Kühnen and Oyserman (2002) might be expected to operate by drawing attention to features that distinguish between me and other, while interdependence priming would draw attention to features that me and other are sharing. As suggested by Hume’s (1739/1978) bundle theory of the self, self-perception (i.e., the current construal of one’s minimal self) would not only be a function of the stimulus features characterizing me and other but also by the attentional weight each feature receives. Accordingly, weighting shared features more strongly would increase the perceived overlap between me and other while a stronger weighting of discriminating features would decrease the overlap. As suggested by Hommel et al. (2009), greater me-other overlap will increase the likelihood that the action of the other is considered in one’s own task representation, which again increases the SSE.

What might be the mechanism responsible for this increase? There is increasing evidence that the SSE is sensitive to both social and non-social factors. For instance, Dolk et al. (2011, submitted) and Dittrich et al. (2012) showed that even non-social events can produce an SSE if they are sufficiently salient. And this is indeed what our theoretical framework suggests: social and non-social events are represented alike, even though there is evidence that social events are more salient and attract more attention (e.g., Friesen and Kingstone, 1998; Langton and Bruce, 2000). Dolk et al. (2011, submitted) suggest that the presence of another salient event in addition to the participant’s own action induces uncertainty about agency, that is, it is no longer clear which of the two events is representing the participant’s own action. Resolving this uncertainty requires the emphasis on features that discriminate between the action of the participant and the action of the co-actor. The most obvious and most salient feature in the standard task set-up is relative location (Guagnano et al., 2010), which means that participants will attend more to, and code more strongly the location of their response (relative to the response of the other), thus creating the SSE. From this perspective, increasing self-other overlap (as by means of interdependence priming) is not the only way to increase the SSE but a particularly effective one.

With regard to cultural variations in the degree of self-other integration, our findings would be consistent with the assumption that culture-specific reward schedules operate on developing individuals. As we have argued elsewhere (Hommel and Colzato, 2010), individuals are likely to acquire preferences for particular control styles through selective reward from their peers. In particular, perceptual, attentional, and action-related processes are under the control of executive functions that specify control parameters (such as speed vs. accuracy, local vs. global processing, or inclusive vs. exclusive decision-making; see Logan and Gordon, 2001; Hommel, 2012), and it makes sense to assume that social reward can bias individuals toward particular ranges of parameter values (Hommel and Colzato, 2010; Hommel et al., 2011). Even though these biases or default values would be acquired in social situations, they are likely to generalize to any situation that is affected by the same executive control function. This would explain why the preference for a high degree of self-other integration in Asian cultures comes along with a more pronounced tendency for integration in non-social perceptual tasks (Boduroglu et al., 2009) and in Social Simon tasks (Colzato et al., 2012).

## Conflict of Interest Statement

The authors declare that the research was conducted in the absence of any commercial or financial relationships that could be construed as a potential conflict of interest.
